# Beyond antipsychotics: a twenty-first century update for preclinical development of schizophrenia therapeutics

**DOI:** 10.1038/s41398-022-01904-2

**Published:** 2022-04-07

**Authors:** Daisy L. Spark, Alex Fornito, Christopher J. Langmead, Gregory D. Stewart

**Affiliations:** 1 Drug Discovery Biology, Neuroscience & Mental Health Therapeutic Program Area, and Neuromedicines Discovery Centre, Monash Institute of Pharmaceutical Sciences, Monash University, 381 Royal Parade, Parkville, VIC 3052 Australia; 2 Turner Institute for Brain and Mental Health, Monash Biomedical Imaging, and School of Psychological Sciences, Monash University, Clayton, VIC 3800 Australia

**Keywords:** Molecular neuroscience, Predictive markers

## Abstract

Despite 50+ years of drug discovery, current antipsychotics have limited efficacy against negative and cognitive symptoms of schizophrenia, and are ineffective—with the exception of clozapine—against any symptom domain for patients who are treatment resistant. Novel therapeutics with diverse non-dopamine D_2_ receptor targets have been explored extensively in clinical trials, yet often fail due to a lack of efficacy despite showing promise in preclinical development. This lack of translation between preclinical and clinical efficacy suggests a systematic failure in current methods that determine efficacy in preclinical rodent models. In this review, we critically evaluate rodent models and behavioural tests used to determine preclinical efficacy, and look to clinical research to provide a roadmap for developing improved translational measures. We highlight the dependence of preclinical models and tests on dopamine-centric theories of dysfunction and how this has contributed towards a self-reinforcing loop away from clinically meaningful predictions of efficacy. We review recent clinical findings of distinct dopamine-mediated dysfunction of corticostriatal circuits in patients with treatment-resistant vs. non-treatment-resistant schizophrenia and suggest criteria for establishing rodent models to reflect such differences, with a focus on objective, translational measures. Finally, we review current schizophrenia drug discovery and propose a framework where preclinical models are validated against objective, clinically informed measures and preclinical tests of efficacy map onto those used clinically.

## Introduction

Schizophrenia is a syndrome commonly associated with symptoms that are classified into positive (psychosis involving hallucinations and/or delusions), negative (affective flattening, anhedonia, avolition), and cognitive (deficits in memory, attention, learning, executive function) domains [1]. Since its emergence in the 1950s and 60s, the dopamine hypothesis has been the leading theory of schizophrenia pathophysiology [2]. While this hypothesis posited that schizophrenia—without specific reference to symptom domains—occurred as a result of excessive neurotransmission at dopamine receptors, it has since evolved to incorporate new lines of evidence [2]. As it currently stands, the dopamine hypothesis proposes that multiple genetic and environmental factors lead to increased presynaptic dopamine function in the striatum, resulting specifically in psychotic symptoms; while reduced dopaminergic drive to cortical areas leads to negative and cognitive symptoms. While the evidence supporting various tenets of this basic model has not always been consistent, dopamine dysregulation has been the target of all drugs used to treat schizophrenia since the discovery of the first antipsychotic, chlorpromazine, nearly 70 years ago [2–4].

Drugs currently approved to treat schizophrenia are generically categorised as either typical or atypical antipsychotics, but all share a common mechanism of action in antagonism of the dopamine D_2_ receptor and numerous issues relating to efficacy. Drugs are effective against positive symptoms in only ~70% of patients (non-treatment resistant, non-TRS; for review see ref. [5]). The remainder recieve no therapeutic benefit from first-line antipsychotics (treatment-resistant schizophrenia, TRS), leaving clozapine as the sole medicinal option—one associated with significant side effects and lifelong monitoring. Moreover, none produce meaningful improvements in negative nor cognitive symptoms—both of which are associated with poor functional outcomes [6–8]. It is evident from both the limited efficacy of current antipsychotics and an array of clinical research that symptoms of schizophrenia occur as a result of diverse brain dysfunction; including changes to a number of neurotransmitter circuitry and immune system function [9–12]. Despite progress towards a better understanding of the underlying pathophysiology, the landscape of schizophrenia therapeutics has not kept pace [6]. The field still awaits a novel drug without appreciable affinity for D_2_ receptors that receives widespread clinical use—and this is certainly not due to a lack of effort.

Therapeutics with novel, non-D_2_ mechanisms have been sought for more than 30 years and include targets as diverse as the receptors, transporters and signalling pathways of dopamine, glutamate, glycine, serotonin, acetylcholine, oxytocin, histamine, opioids and neurosteroids [6]. Compounds for these targets have often shown promising efficacy in preclinical development, mostly in rodents, but consistently fail in clinical trials due to a lack of efficacy. A recent review of 250 clinical trials of drugs with non-D_2_ mechanisms concluded *“…we cannot confidently state that any of the mechanistically novel experimental treatments covered in this review are definitely effective for the treatment of schizophrenia and ready for clinical use.”* [6].

While the large number of failed clinical trials may be attributed to shortcomings at multiple stages in the drug discovery process, there is a clear and systematic disconnect between preclinical and clinical efficacy—particularly with respect to negative and cognitive symptoms [13, 14]. This may be unsurprising given the disparate measures by which efficacy is assessed in rodents vs. humans. In clinical trials, various clinical rating scales or cognitive batteries are used to quantify efficacy of schizophrenia therapeutics against positive, negative and cognitive symptoms [15, 16]. Clearly, interview-based clinical rating scales cannot be used in rodents, therefore preclinical efficacy is assessed using behavioural tasks that act as correlates of human symptoms, such as rodent locomotor activity and positive symptoms. While the ultimate goal of assessing efficacy in preclinical models is to provide a prediction of clinical efficacy, there are limited instances in which preclinical efficacy does indeed correlate with clinical efficacy—likely attributed to not only species differences but disparate methods of assessment.

In an industry that increasingly strives to test new drugs in a “fail fast” approach, the inability to reliably predict clinical outcomes poses a significant and costly challenge [17]. Without a critical evaluation and subsequent reimagination of preclinical discovery, it is unlikely such challenges will be overcome. Here, we discuss the past and present landscape of schizophrenia drug development, particularly where the efficacy of novel non-D_2_ candidates has failed to translate in late-stage clinical trials, then highlight key issues in preclinical development limiting accurate predictions of clinical efficacy. Finally, we consider a revised framework whereby neuroimaging could be used as a translational tool to improve preclinical predictions of clinical outcomes.

## The past and the present of schizophrenia drug development

It is clear that current antipsychotics have limited utility in treating the broad spectrum of symptoms in all schizophrenia patients, thus novel non-D_2_ therapeutics have been sought with the idea that they will treat all symptom domains and be effective in TRS patients. Disappointingly, there has been an overwhelming failure to convert promising preclinical data, and in some cases encouraging early clinical findings, into successes in phase 3 and new drug approvals for the treatment of symptom domains of schizophrenia. Inhibitors of PDE10 (e.g. PF-02545920 and TAK-063), that act downstream of dopamine signalling, have failed to show a significant improvement in PANSS total score in Phase II clinical trials despite showing D_2_ antagonist-like activity in preclinical models [18, 19]. Similarly, drugs that act to enhance glutamate signalling at either NMDA (e.g. glycine transporter type-1 inhibitor bitopertin and D-amino acid oxidase inhibitor luvadaxistat) or metabotropic glutamate (e.g. mGlu2/3 agonists such as pomaglumetad) receptors have failed to meet primary endpoints in mid-to-late-stage clinical development for negative and cognitive symptoms, despite promising preclinical data [20–23]. Alpha-7 nicotinic acetylcholine receptor agonists (e.g. encenicline), which have been shown to modulate dopamine, glutamate, and acetylcholine, displayed promising efficacy against cognitive symptoms in a Phase II clinical trial but again failed due to efficacy in two Phase III trials [24, 25].

The aetiology of these failures is unclear; collectively therapeutics targeting solely the glutamatergic system alone appear to have fared poorly in later-stage clinical trials. However, both glycine transporter type-1 inhibitors (e.g., BI-425809) and AMPAkines (e.g., PF-04958242, now BIIB104) remain in active mid-late-stage clinical development for different symptom domains in schizophrenia.

AMPAkines are positive allosteric modulators of AMPA receptors and act to enhance long-term potentiation, a critical process involved in learning and memory formation [26]. The first AMPAkine to be clinically tested for schizophrenia, CX516, was assessed as a possible add-on therapy to standard of care antipsychotic drugs [27]. However, CX516 worsened PANSS scores compared with the placebo group and did not improve patient cognitive outcomes. By virtue of their mechanism of action, AMPAkine clinical trials have largely investigated improvements to cognition—it is unclear whether efficacy may extend to positive or negative symptoms. AMPAkines improve cognition in preclinical cognitive deficit models, thought to be through the enhancement of long-term potentiation and long-term depression [26]. BIIB104 also improved ketamine-induced cognitive deficits in healthy subjects in the Hopkins verbal learning test, however this may be confounded through the activity of ketamine in potentiating AMPA receptor activity [28, 29]. It remains to be seen whether these results will predict efficacy in late-stage clinical development.

Furthermore, the endogenous glutamate tone will likely play a key role in any clinical efficacy. While TRS patients exhibit normal levels of striatal dopamine compared to treatment-sensitive patients, they express a higher concentration of cortical glutamate; therefore it is difficult to predict the extent of AMPA receptor potentiation by an AMPAkine [30]. It is also unclear how synaptic glutamate levels change as the disease progresses and during exacerbation of symptoms.

With respect to non-glutamatergic approaches, despite many similarities with the phenotype of dopamine D_2_ receptor antagonists in preclinical studies, multiple PDE10 inhibitors failed due to lack of efficacy in clinical development. Recent speculation attributes failures of such inhibitors to their only modulating indirect spiny projection neurons in the striatum and / or the additional effects on cyclic GMP with respect to modulation of cyclic AMP by dopamine D_2_ receptor antagonists [31].

Irrespective, the overall picture is one of a disconnect between preclinical testing and clinical evaluation. Thus, it is with cautious optimism that recent positive phase 2 clinical data with KarXT (a combination of the muscarinic acetylcholine receptor agonist, xanomeline, and the peripherally-restricted agonist, trospium) and ulotaront (TAAR1 agonist; SEP-363856) provides potential hope for alternative, non-dopamine D_2_ receptor-based medicines.

In a four-week phase 2 trial in patients with acute exacerbation of schizophrenia, the TAAR1 agonist, ulotaront, significantly improved PANSS total scores with respect to placebo control, with a generally favourable side effect profile (including a lack of metabolic effects) [32]. It remains to be seen whether this efficacy persists after longer term treatment. TAAR1 agonists are proposed to exert antipsychotic efficacy by dampening dopamine signalling in the striatum, through presynaptic inhibition of dopaminergic neuronal firing, dopamine release and/or synthesis capacity [33]. Given the proposed mechanism, it is unlikely that ulotaront would be effective in TRS patients, who do not display increased presynaptic dopamine function [34]. Behavioural data from preclinical animal models suggests ulotaront may also possess efficacy against negative and cognitive symptoms, however this is yet to be confirmed in a clinical setting [35, 36].

KarXT also recently completed a five-week phase 2 trial in inpatients with established schizophrenia, improving PANSS total, positive and negative scores with respect to placebo control [37]. These data complemented prior studies in which xanomeline was effective versus PANSS total scores and reduced psychosis-like behavioural disturbances in Alzheimer’s disease patients [38, 39]. Preclinical data suggest that it is primarily the M_4_ receptor agonist activity (rather than the M_1_ activity) that engenders the antipsychotic effect, which would be consistent with the recently reported antipsychotic activity of CVL-231, a subtype selective positive allosteric modulator of the M_4_ receptor, in a phase 1b trial [40–42].

The presynaptic expression of the inhibitory M_4_ receptor on cortical glutamatergic afferent terminals in the striatum (as well as post-synaptically on dopamine D_1_-expressing spiny projection neurons), presents the enticing theory that activation of the M_4_ receptor might be an effective treatment in TRS. Whereas there is unchanged striatal presynaptic dopamine content in TRS (thus no hyperdopaminergia to normalise), there are elevated anterior cingulate cortical glutamate levels, which may increase corticostriatal drive, and be normalised by M_4_ receptor activation. Furthermore, both M_1_ and M_4_ receptor activation could yield pro-cognitive efficacy; thus the clinical benefit of KaXT and those compounds in its wake will be interesting to follow [43, 44].

## Determinants of preclinical efficacy

Despite disappointing results in humans, each novel therapeutic discussed in the previous section showed promise in preclinical development. Preclinical assessments of efficacy can be broken down into two components: models and tests. Models refer to the methods by which schizophrenia-relevant behaviours are induced in laboratory animals, while tests quantify the magnitude and robustness of schizophrenia-relevant changes in these models, and thereafter the extent to which a novel therapeutic can reverse them. The scope of available animal models and tests has been reviewed extensively elsewhere (see ref. [45]); this section will instead serve as a primer on the challenges that likely contribute to the lack of translation between preclinical and clinical efficacy. First, it must be emphasised that schizophrenia is inherently a human disorder. As such, there will be an unavoidable limit to which animal models are relevant to human symptoms and can therefore predict clinical outcomes. Given the challenges outlined below, the utility of animal models has not yet been exhausted.

## Preclinical models

Schizophrenia-relevant models can be produced by a variety of interventions, commonly in mice and rats, broadly characterised as genetic, pharmacological, and neurodevelopmental models [45]. While some genetic models have been developed in response to hypotheses of schizophrenia pathophysiology (e.g., dopamine transporter knockout or NMDA receptor NR1 subunit knockdown), others reflect findings from genetic studies of schizophrenia patients (e.g., DISC1, 22q11.2 deletion and NRG1 models) [46, 47]. The latter have been somewhat limited by the heterogeneous and polygenic nature of schizophrenia. Polygenic risk scores derived from GWAS studies are strongly associated with schizophrenia, however reverse translating this genetic liability to animal models is likely impossible given the complexity, even before addressing genetic differences between humans and rodents [48]. Pharmacological models also reproduce theories of neurotransmitter dysfunction: amphetamine induces striatal hyperdopaminergia while NMDA antagonists such as phencyclidine and ketamine reflect NMDA receptor hypofunction. Acute and sub-chronic dosing produces behavioural effects reflective of a psychotic episode and chronic illness, respectively. Neurodevelopmental models (e.g., methylazoxymethanol acetate, maternal immune activation, post-weaning social isolation) have been developed to recapitulate certain risk factors for schizophrenia, and perhaps provide some of the most appropriate models with respect to schizophrenia aetiology, particularly when combined in multiple-hit models.

To assess the validity of each model with respect to human illness, a set of criteria have been established [49]:

1. Face validity: model reflects rodent correlates of positive, negative and cognitive symptoms
2. Construct validity: behavioural dysfunction is a result of neurochemical and structural alterations similar to those present in the brains of schizophrenia patients
3. Predictive validity: therapeutic effects in the model are predictive of efficacy in humans.

No current rodent model meets the criteria for face, construct, and predictive validity. Schizophrenia is an exclusively human disorder that is highly heterogeneous with respect to symptoms, pathogenesis and aetiology; therefore, it is highly unlikely that an animal model meeting all criteria for face, construct and predictive validity will ever be established. As such, preclinical testing is often spread across a variety of models to ensure varying aspects of face and construct validity are appropriately met.

Early animal models of schizophrenia were based on the observation that dopamine enhancing drugs, such as amphetamine, produce psychotic symptoms in healthy individuals and worsen positive symptoms in schizophrenia patients [50, 51]. Dopamine-centric models were further solidified by the finding that drugs with antipsychotic efficacy antagonise the dopamine D_2_ receptor, for which their affinity is highly correlated with clinical efficacy [52]. While dopamine models do not reflect the complex interaction between genetic and environmental factors that contribute to schizophrenia pathophysiology and therefore have limited construct validity, they have had relative success with respect to their predictive validity, specifically with regard to treating psychosis in schizophrenia. However, this strategy has only proven useful for drugs that perturb the increase in dopaminergic neurotransmission produced by such models [49].

Early theories of dopamine dysfunction in schizophrenia are further embedded in schizophrenia drug discovery with the use of current antipsychotics to validate rodent models. The limitations of this approach are twofold: First, selecting models based on the efficacy of drugs with a common mechanism of action severely limits the model’s ability to identify drugs with distinct mechanisms of action. This is largely problematic as current drugs are ineffective, with the exception of clozapine, in ~30% of patients [5]. Second, antipsychotics do not produce meaningful improvements in negative or cognitive symptoms. Therefore, using such drugs to validate models may create a bias away from clinically relevant representations of negative and cognitive symptoms.

Finally, it is important to note the reciprocal relationship between preclinical models and the behavioural tests used to validate such. The basic tenet is that disrupted behaviours in various animal models are more likely relevant to schizophrenia than behaviours that are unaffected by such interventions [53]. As such, these disrupted behaviours inform the tests that are used to assess correlates of human symptoms and the measures by which new models are validated. For example, early dopamine- and glutamate-based pharmacological rodent models yield increased locomotor activity, which is now commonly used as a correlate of positive symptoms to assess antipsychotic efficacy and validate new models. To this extent, models and tests select for each other in a self-reinforcing loop, conceivably creating a bias away from schizophrenia-relevant features.

## Preclinical tests

As alluded to in the previous section, the reliance on behavioural paradigms to assess human symptoms presents a significant limitation of preclinical testing. Rodents are not prone to perceptual disturbances such as hallucinations and delusions, therefore positive symptoms are generally assessed by an increase in locomotor activity and stimulant sensitivity [54]. Although there is an obvious disconnect between hyperactivity and paranoid ideations, a reduction in locomotor activity has been used as a correlate of antipsychotic efficacy with relative success for D_2_ antagonists [55]. However, while all antipsychotics reduce locomotor activity—whether in response to stimulant, stress or a novel environment—not all drugs that reduce locomotor activity possess antipsychotic efficacy [55]. The latter suggests that the efficacy of current antipsychotic drugs against positive symptoms is reflective of a conserved mechanism of action, rather than a test that accurately assesses behaviour related to psychosis. Notwithstanding, circuit-level approaches and the recent description of dopamine-mediated hallucination-like perception in mice represent progress towards objective assessment of positive symptoms in rodents [56, 57].

A similar disconnect is also apparent in the preclinical tests used to assess negative and cognitive symptoms; however, these methods are not predictive for discriminating drugs with human efficacy. While the serendipitous discovery of chlorpromazine provided a prototype for drugs that reverse positive symptoms, and therefore a behavioural profile in rodents indicative of clinical efficacy, there is yet to be a drug that produces meaningful improvements in negative and cognitive symptoms. As such, there is no benchmark for clinically relevant changes to correlates of negative and cognitive symptoms in rodents.

Negative symptoms are typically assessed in rodents by reduced social interaction and reduced preference or motivation for palatable substances [54]. Although these tests are attractive as they are relatively simple and conceptually align with negative symptoms in schizophrenia patients, without a complete understanding of the physiological mechanisms that lead to these behavioural changes it is unclear how relevant they are to human disease. For example, olfaction plays a large role in the social behaviour of rodents, therefore it is possible that genetic or pharmacological manipulations that alter social behaviours do so via disrupted olfactory processing rather than changes in cognitive pathways common to patients [58]. While there is evidence of disrupted olfactory processing in schizophrenia, it is unlikely that this contributes to changes in social behaviours to the extent it does in rodents [59].

Importantly, the extent of cognitive deficits in humans better predicts patient outcomes than either positive or negative symptoms, yet are untreated by current antipsychotic drugs [45, 60–62]. Measures of pro-cognitive efficacy in rodents following treatment with putative schizophrenia therapeutics have been notoriously poor at translating into clinical efficacy. Preclinical cognitive tests frequently identify drugs with nootropic activity in rodents, but are unable to determine those with potential for efficacy in humans [13, 14]. Traditional methods of evaluating pro-cognitive efficacy in rodents include the Morris water maze, T-maze, radial arm maze, novel object recognition, 5-choice serial reaction time task and attentional set-shifting tasks, which variously assess cognitive components of memory, attention and executive function [54]. Much like negative symptoms, traditional cognitive assessments are attractive as they are relatively simple and quick to complete, however these tasks do not reliably predict clinical outcomes following treatment with putative schizophrenia therapeutics. This is highlighted by the findings that acetylcholinesterase inhibitors, α7 nAChR partial agonists, AMPA (GluA)-positive allosteric modulators, GABA_A_ α2/α3 partial agonists, and DAT and NET inhibitors reverse cognitive dysfunction in a putatively schizophrenia-relevant rodent model as determined by the novel object recognition task, yet do not improve cognition, as determined by a range of rating scales, in clinical trials of schizophrenia patients (Table 1) [27, 63–75]. Traditional tasks adapted from human counterparts offer no better predictions of clinical efficacy, suggesting that as a whole these methods are not stringent enough to identify clinically meaningful changes in cognition.

**Table 1:** Investigational schizophrenia therapeutics with nootropic efficacy in the rodent novel object recognition task do not possess clinical pro-cognitive activity.

Investigational drug	Mechanism of action	Clinical efficacy
Donepezil	AChE inhibitor	Not significantly different from placebo
		Determined by CATIE neurocognitive battery
GTS-21/DMXB-A	α7 nAChR partial agonist	Not significantly different from placebo
		Determined by MCCB
CX516	AMPAR PAM	Not significantly different from placebo
		Determined by cognitive battery (similar to MCCB)
MK-0777/TPA-023	GABA_A_R α2/α3 partial agonist	Not significantly different from placebo
		Determined by MCCB
Modafinil	DAT inhibitor	Not significantly different from placebo
		Determined by COGBAT
Atomoxetine	NET inhibitor	Not significantly different from placebo
		Determined by BACS

*AChE* acetylcholinesterase, *AMPAR PAM* α-amino-3-hydroxy-5-methyl-4-isoxazolepropionic acid receptor positive allosteric modulator, *BACS* Brief Assessment of Cognition in Schizophrenia, *CATIE* Clinical Antipsychotic Trials of Intervention Effectiveness, *DAT* dopamine transporter, *GABAA* gamma-aminobutyricacid A receptor, *MCCB* MATRICS Consensus Cognitive Battery, *nAChR* nicotinic acetylcholine receptor, *NET* norepinephrine transporter

More recently, the development of rodent touchscreen paradigms has begun to bridge the translational gap between assessments of rodent and human cognition [76]. Touchscreen tasks can probe a range of cognitive processes including reward learning, memory, perceptual discrimination, object-place associative learning, attention, impulsivity, compulsivity, extinction and simple Pavlovian conditioning within the same chamber, thereby providing context independence and a tightly controlled assessment of broad cognitive function [76]. Critically, it has allowed for the assessment of cognitive processes in rodents and humans in a remarkably similar manner [77, 78]. The translational power of this is demonstrated by the finding that *Dlg2* knockout mice and humans with *DLG2* CNV deletions, reported in schizophrenia patients, show strikingly similar impairments in cognitive function determined by an identical object-located paired associates task [77, 79].

While these results are certainly promising for the future of pro-cognitive drug development, touchscreen assessments do not go without limitations. Establishment costs and the time required to complete touchscreen tasks—some of which can take up to three months or more—mean traditional assessments of rodent cognition are still favoured in schizophrenia drug discovery. Nonetheless, the fast turnaround for results required in early screens of pro-cognitive efficacy may be possible using touchscreen tasks in the future with the refinement of training periods and development of shortened tasks [80, 81]. A further limitation, although not specific to touchscreen tasks, is the use of food for positive reinforcement of touchscreen performance and the requirement of food restriction for this to be effective. Any change in motivation towards the reward, either by drug treatment or preclinical model, will impact task performance but not necessarily through the same mechanisms as in humans who do not require a food reward. In addition, food restriction alone is shown to have pro-cognitive effects, which may reduce the window for detecting pro-cognitive activity of novel therapeutics [82].

## A new way forward: reverse translation of clinical research

Animal models of schizophrenia are vast and varied, each reflecting some dysfunctional aspects of schizophrenia better than others. As such, it becomes difficult to evaluate novel schizophrenia therapeutics when there is no gold-standard model, and no clear relationship between preclinical and clinical efficacy. This difficulty is compounded by the lack of established biomarkers of schizophrenia; limiting the ability to accurately diagnose, discriminate subtypes, and predict treatment responses in a clinical setting. Nonetheless, the application of non-invasive neuroimaging modalities in clinical research has allowed for unprecedented access into the brains of living schizophrenia patients, alongside significant progress towards disease biomarkers [83, 84].

**Fig. 1:**
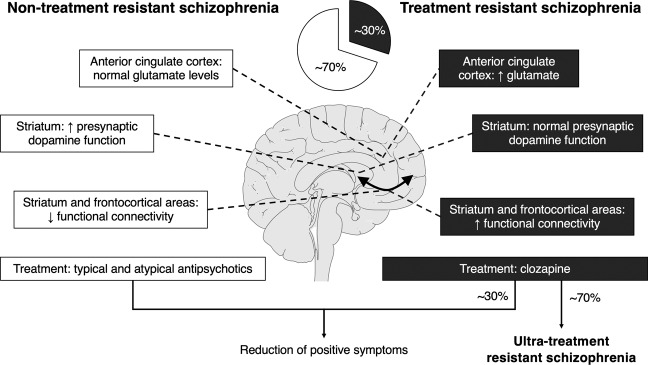
Neurobiology of nontreatment-resistant and treatment-resistant schizophrenia. Approximately 30% of schizophrenia patients do not respond to first-line antipsychotics; treatment response or non-response is associated with distinct neurobiological changes within the corticostriatal circuitry. Of treatment-resistant patients, approximately 70% do not respond to clozapine. No treatment provides meaningful improvements to negative or cognitive symptoms.

Thousands of published studies report diverse structural and functional changes in patients, with meta-analyses providing some clues as to which disruptions may be more prominent [85]. Perhaps of greatest relevance to preclinical drug discovery is the finding that schizophrenia patients who do not respond to first-line antipsychotics are neurochemically distinct from treatment-responsive patients (Fig. 1) [34, 86–88]. Treatment-resistant schizophrenia is generally defined as ≥2 periods of treatment with different antipsychotics at a therapeutic dose equivalent to ≥600 mg chlorpromazine per day, each for at least 6 weeks, without at least a 20% reduction in symptoms [89]. There are two distinct presentations of treatment resistance: one that is present from illness onset and another that develops over the course of illness, with the majority (70–84%) of patients falling into the former category [90, 91]. While such distinction has clear clinical relevance—clozapine is the only drug indicated for use in treatment-resistant patients—it is rarely recognised at the preclinical level. Here, we consider the potential of using human neuroimaging research to guide preclinical efforts, with a specific focus on findings that distinguish treatment-resistant and treatment-responsive subtypes of schizophrenia [12].

## Dopamine-mediated dysfunction of corticostriatal circuits

Dopamine dysfunction has been the longest standing theory of schizophrenia pathophysiology, emerging in the mid-1900s from the observation that drugs that increase extracellular dopamine, such as amphetamine, induce psychotic symptoms in healthy subjects and worsen those in schizophrenia patients [92]. The involvement of dopamine was further supported with the discovery that drugs effective at treating psychosis, such as reserpine, chlorpromazine and haloperidol, dampen dopaminergic tone; antipsychotic efficacy was later found to be directly correlated with affinity for dopamine receptors [52, 93–96]. In the decades since, molecular neuroimaging studies have shed significant light on the exact nature of dopamine dysfunction in vivo.

Positron emission tomography (PET) has proven to be a versatile tool for the assessment of multiple aspects of dopamine function in vivo, including dopamine receptor and transporter availability, and capacity for dopamine synthesis and release [97]. While some findings are inconsistent across studies (likely reflecting the heterogeneous nature of schizophrenia), arguably the most robust indicator of dopamine dysfunction in schizophrenia patients is that of increased presynaptic dopamine function, which can be indexed by a number of measures [98, 99]. Dopamine synthesis capacity is quantified by the rate of uptake of radiolabelled L-DOPA (^18^F-DOPA) in the striatum, where it is converted to ^18^F-dopamine by aromatic L-amino acid decarboxylase, therefore providing a complex measure of presynaptic dopamine function [100, 101]. Additionally, D_2_ receptor availability can be assessed at baseline or following dopamine release or depletion using D_2_ PET tracers [99].

Meta-analyses of striatal dopamine synthesis capacity or presynaptic dopamine function more broadly have found both to be significantly greater in schizophrenia patients than healthy controls, with large effect sizes (d’) of 0.867 and 0.79, respectively [98, 102]. Moreover, the increase in striatal dopamine synthesis capacity was not influenced by subject age or gender, year of publication, duration of illness, psychotic symptoms or antipsychotic exposure [102]. While there is strong evidence to suggest that increased presynaptic dopamine function is a core pathophysiological feature for a large proportion of schizophrenia patients, it is also apparent that there is a proportion of patients for whom this is not the case. Here the distinction between treatment-responsive and treatment-resistant patients is critical: those diagnosed as treatment resistant (TRS) have significantly lower dopamine synthesis capacity than patients who respond to first-line antipsychotics (non-TRS) [34, 87, 103, 104]. In some instances, dopamine synthesis capacity in TRS patients was found to be comparable to healthy controls, adding weight to the argument that patients can be neurochemically different yet have a similar disease manifestation [34, 103].

Together with converging lines of evidence from studies of dopamine metabolites and density of striatal dopaminergic synapses, it has been proposed that schizophrenia may be further classified into subtypes based on the status of dopamine function [12]. Namely, hyperdopaminergic patients who show a reduction in symptoms following treatment with first-line antipsychotics, and normo-dopaminergic treatment-resistant patients that may only benefit from treatment with clozapine, if at all [12, 105]. This distinction has significant implications for preclinical schizophrenia drug discovery, particularly in the context of developing effective pharmacological interventions for TRS. It is for this reason that any clinical differentiation between KarXT and ulotaront (*vide supra)* will be interesting to follow as they both move towards late-stage development.

Consequences of elevated or normal presynaptic dopamine function in non-TRS and TRS, respectively, have been observed more broadly with respect to changes in corticostriatal functional connectivity. The idea of dysconnectivity in schizophrenia has been implicit since conception, but it was not until the advent of non-invasive neuroimaging techniques in the 1980s and 1990s that there was clear evidence to support pathophysiological disconnection of widespread neural systems [106–108]. Functional magnetic resonance imaging (fMRI), which uses localised signal disturbances caused by the magnetic susceptibility of deoxyhemoglobin as a proxy for neuronal activity, has been particularly useful in delineating schizophrenia-related changes in connectivity. Inferences of functional connectivity are derived by the statistical association of activity between spatially distinct regions, either during task or in spontaneous dynamics recorded during task-free, so-called “resting” states [109].

Interestingly, patients often show disrupted functional connectivity within corticostriatal systems known to be influenced by dopamine, and it appears that presynaptic dopamine function, corticostriatal connectivity, and treatment response are inextricably linked [104, 110, 111]. In a recent combined PET and resting-state fMRI study, the relationship between presynaptic dopamine function and corticostriatal functional connectivity was found to differ between TRS and non-TRS patients. An inverse relationship between dopamine synthesis capacity and strength of connectivity between the associative striatum and ventromedial prefrontal cortex was found in TRS patients, but not in non-TRS patients or healthy controls [104]. Notably, the difference between presynaptic dopamine function in TRS patients and healthy controls was greatest in the associative striatum [112].

While not investigating dopamine-related changes directly, divergent patterns of functional connectivity in TRS and non-TRS patients provides further support for subtype-dependent changes to corticostriatal circuitry [83, 113, 114]. With respect to healthy controls, TRS patients have reduced connectivity between the ventral striatum and the middle frontal gyrus, and between the dorsal striatum and sensorimotor cortex, whereas non-TRS patients show reduced connectivity between the dorsal striatum and both the dorsolateral PFC and visual cortex [114]. Furthermore, TRS patients exhibit greater connectivity between the dorsal striatum, including the associative striatum, and medial prefrontal cortex than non-TRS patients [114]. Finally, in a study investigating striatal connectivity as a putative biomarker of treatment response, striatal connectivity with frontal cortical regions, including orbitofrontal and anterior cingulate cortices, was found to be a negative predictor of treatment response [83]. Together this suggests a pattern of hypofrontostriatal connectivity in TRS and hyperfrontostriatal connectivity in non-TRS patients.

Given that TRS patients have normal presynaptic dopamine function, it stands to reason that symptoms and corticostriatal dysconnectivity arise from dysfunction elsewhere. It has been proposed that maladaptations may be present at other levels of the dopaminergic system, including increased expression of D_2_ receptors and increased sensitivity of D_2_ receptors to dopamine [115, 116]. Beyond dopamine, magnetic resonance spectroscopy (MRS) has proven useful for identifying neurochemical changes in the brains of schizophrenia patients in a non-invasive manner. With respect to TRS, there is increasing evidence to suggest that elevated glutamate in the anterior cingulate cortex (ACC) is a distinguishing feature of this subtype [117]. Although varying definitions of treatment resistance likely contribute to some inconsistencies between studies, a majority report elevated levels of glutamate in the ACC of TRS patients, with respect to non-TRS patients or healthy controls [30, 118–121]. A potential caveat to such findings is the effect of antipsychotic treatment and age on ACC glutamate [122, 123]. However, in all instances TRS and non-TRS patients were matched for age and medication status, therefore providing some confidence that divergent changes to ACC glutamate are associated with treatment response, or lack thereof. Interestingly, there is an inverse relationship between cortical glutamate and striatal dopamine, suggesting that differences in presynaptic dopamine function and ACC glutamate between subtypes may also be linked [124, 125].

## Reverse translation to animal models: promises and challenges

A vast number of neurodevelopmental, pharmacological, and genetic rodent models exist, each with varying degrees of relevance to human disorder. Clinical neuroimaging research has provided strong evidence to suggest that treatment response in schizophrenia patients, or lack thereof, is due to distinct neurochemical profiles (Fig. 1) [12, 104]. These differences have significant implications for not only the course of treatment, but the way in which new therapeutics are developed. Without an established animal model of TRS, it is unsurprising that clozapine remains the only approved pharmacological treatment for this subtype. Residual symptoms can persist in TRS even after initiation of clozapine treatment, therefore it is critical that novel therapeutics for TRS patients are assessed in a model that reflects their distinct pathophysiology [86, 126]. Conversely, assessing preclinical efficacy of drugs for non-TRS in models that reflect underlying changes to neurobiology may improve predictions of clinical efficacy [127]. In this section we propose criteria for the validation of TRS- and non-TRS-relevant models, discuss the importance of establishing such models, and outline challenges that lie ahead.

By the proposed criteria, TRS and non-TRS models are distinguished by presynaptic dopamine function, anterior cingulate glutamate concentration and corticostriatal functional connectivity (Table 2). It may appear counterintuitive to omit behavioural response to first-line antipsychotics from the proposed criteria, given that this is the very crux of treatment-responsive and -resistant subtypes of schizophrenia. However, the lack of relationship between preclinical and clinical efficacy suggests that antipsychotic response (e.g. a reduction in psychostimulant-induced hyperactivity) may not be a robust measure of treatment resistance in rodents. Nonetheless, assessing antipsychotic response in models meeting the above criteria will be an important step towards clarifying the relationship between behavioural measures used as symptom correlates and underlying pathophysiology.

**Table 2:** Criteria for treatment-resistant and non-treatment-resistant models.

Translational measure	Non-treatment resistant	Treatment resistant
Presynaptic dopamine function assessed by PET	Elevated	Normal
Glutamate in anterior cingulate cortex assessed by MRS	Normal	Elevated
Corticostriatal functional connectivity assessed by fMRI	Reduced connectivity between striatum and frontal cortical areas	Increased connectivity between dorsal striatum and medial prefrontal cortex

A particular strength of the proposed criteria is the highly translational measures by which TRS and non-TRS models are defined. While rodent models are limited by the fact that schizophrenia is an exclusively human disorder, the disparate measures by which symptoms, and a reduction thereof, are assessed in preclinical models vs. patients presents an additional hurdle for translating preclinical to clinical efficacy. Clinical neuroimaging modalities such as PET, MRS and fMRI are increasingly used to investigate rodent models of brain disorders, with the recognition that these techniques may act as objective translators of pathophysiology between species [128–130]. Although the translational potential has long been recognised, there is still relatively little known about schizophrenia models with respect to how accurately they recapitulate findings from clinical neuroimaging studies of schizophrenia patients [131]. Consequently, these techniques are seldom used to assess preclinical activity. While it is certainly true that the poor uptake is due, at least in part, to the historical limitations of these techniques in much smaller rodent brains and specialist equipment, it may be further explained by the difficulty in rationalising which of the vast findings from clinical research are critical to recapitulate in rodent models, and the uncertainty around how doing so will measurably improve the drug discovery process.

Here, the application of neuroimaging in preclinical discovery is reconciled in a way that serves an objective goal: to establish TRS and non-TRS models of schizophrenia in order to improve predictions of clinical outcomes in each subtype. We propose a two-step application of the criteria: first, to revalidate existing models as models for TRS and non-TRS, rather than the umbrella schizophrenia. Second, where existing models fall short, new models should be developed specifically to meet the translational criteria for TRS and non-TRS. The EDiPS model provides a recent example of this, which has been developed specifically to recapitulate elevated presynaptic dopamine function found in non-TRS patients [132]. Behavioural validation will still be important, particularly for negative and cognitive symptoms, however focusing on translational validation criteria as a first pass may skew the models towards more clinically relevant representations of illness. This does not preclude the existence of other neurobiological subtypes—models for such should be developed pending strong clinical evidence.

As history has shown, identifying and defining disease subtypes has a significant impact on treatment outcomes [133–135]. The distinction between TRS and non-TRS in preclinical development may indeed serve as a tipping point towards the discovery of next generation schizophrenia therapeutics. Recent work by Kokkinou et al. provides an elegant example of the translational power of using neuroimaging to investigate changes in presynaptic dopamine function associated with non-TRS [130]. The authors find that sub-chronic ketamine treatment in mice produces elevated presynaptic dopamine function, characteristic of non-TRS patients, which is normalised following acute treatment with ulotaront/SEP-363856, a novel TAAR1 agonist schizophrenia therapeutic with a non-D_2_ mechanism of action [35, 130]. Notably, ulotaront has shown efficacy in a recent Phase II clinical trial and extension study with a non-TRS cohort, demonstrating a link between the rodent model and human illness [32, 127]. It is yet to be shown whether ulotaront treatment also normalises presynaptic dopamine function in non-TRS patients, and whether doing so translates to an improved therapeutic profile—particularly with respect to negative and cognitive symptoms—versus current drugs that have no effect on presynaptic dopamine function. Provided that ulotaront normalises presynaptic dopamine function in non-TRS patients, such translational markers may offer an objective assessment of preclinical efficacy, and facilitate the discovery of next generation schizophrenia therapeutics with novel non-D_2_ mechanisms of action. While the above is certainly promising for non-TRS patients, a critical next step will be the application of this strategy in the development of novel treatments for TRS.

Despite the progress to be made from reverse translating the clinical neuroimaging findings discussed above, a number of caveats follow. First, models that meet the proposed criteria for TRS may only be representative of the 70–84% of patients who are deemed TRS from illness onset, but not those who develop TRS over the course of illness [91]. With respect to the latter, the proposed measures may reflect not just treatment response but also chronicity or maladaptations in response to repeated antipsychotic exposure [90]. Without further clinical research to confirm the specificity of the proposed measures to treatment response in patients who develop TRS over the course of illness, it is unclear how predictive TRS models will be for this subset of patients. Second, TRS is defined by a lack of improvement in positive symptoms following antipsychotic treatment and does not address negative and cognitive symptoms. As such, it is unclear whether models meeting the proposed criteria for TRS or non-TRS will offer any advantage with respect to predicting efficacy of schizophrenia therapeutics against negative and cognitive symptoms.

Finally, it is unclear the extent to which the translational power of these techniques will be bound by fundamental differences between rodent and human brains. A recent fMRI study in both mice and humans has begun to address this overarching question, identifying similar patterns of striatal functional connectivity in regions corresponding with limbic and sensorimotor, but not associative circuitry [136]. This finding may be unsurprising given that in humans, the associative striatum receives projections from prefrontal cortical regions involved in executive, social- and language-related functions; cognitive processes that are thought to have developed in primates and humans due to evolutionary pressures [137, 138]. Here, we must reconcile the inherent differences between rodent and human brains with the critical role rodent models play in the earlier drug discovery stages—one that cannot be easily replaced by more homologous species, such as non-human primates, due to ethical and financial considerations. By using translational techniques and therefore replacing current technical limitations with those of species differences, we become much closer to recognising the true potential of rodent models. If we are to succeed in developing effective translation of novel psychiatric medicines it must be through thoroughly benchmarked, standardised and objective measures.

## Conclusions

The past 50+ years of schizophrenia drug discovery has seen little in the way of treatments that effectively manage the disorder: current therapeutics have limited efficacy against negative and cognitive symptoms, and are ineffective against any symptom domain, with the exception of clozapine, in approximately 30% of patients [5]. Paradoxically, our understanding of schizophrenia pathophysiology has significantly progressed during this time: it is now clear that dysfunction occurs across multiple neurotransmitters and brain regions, and that treatment response, or lack thereof, is associated with distinct neurochemical profiles (Fig. 1) [12, 104]. While this knowledge has been applied in drug discovery, a common problem for drugs targeting affected neurotransmitters is the lack of translation between preclinical and clinical efficacy. This repeated roadblock highlights a systematic failure in the methods that determine efficacy in animal models. Evaluation of current methods identifies a number of considerable challenges: rodent models that do not recapitulate all aspects of human disorder, rodent tests of efficacy that have questionable correlation with human symptoms, and the inextricable link between early dopaminergic theories of schizophrenia pathophysiology and the methods by which preclinical efficacy is now determined. None of these challenges are new, yet none have been addressed at an adequate scale involving industry and academia. Engagement of both parties will be critical for not only innovative solutions, but widespread adoption of such to move away from outdated and unreliable methods of determining preclinical efficacy.

Equipped with a growing number of translational techniques and an understanding of the pathophysiology underlying treatment response and non-response, we now see a pivotal opportunity to redefine preclinical drug discovery for the twenty-first century. Here, we outline fundamental principles guiding a roadmap forward. First, rodent models should be validated against objective, clinically informed measures with a greater emphasis on construct validity. Given the distinct neurochemical profiles of TRS and non-TRS patients, validation should also seek to distinguish TRS and non-TRS rodent models. Key experiments will include the re-validation of commonly used rodent models with respect to presynaptic dopamine function, ACC glutamate content and corticostriatal connectivity; in addition to cognitive fingerprinting with a battery of touchscreen-based tasks.

Second, preclinical tests of efficacy should resemble, to the extent permitted by species differences, clinical tests of efficacy. Where clinical tests are unsuitable for rodent adaptation (e.g. symptom rating scales), theragnostic biomarkers may offer more robust measures of efficacy compared to current behavioural-based tasks. A key checkpoint in developing translational measures of efficacy—particularly those for cognition—will be to understand the false-positive rate, i.e., how many drugs without pro-cognitive efficacy in humans are misclassified as pro-cognitive using these tasks? Given this indication will be model-dependent, it is imperative that models are first refined to reflect the current understanding of schizophrenia pathophysiology before judgements about predictive validity are made. Once established, these frameworks must be benchmarked using clinically relevant drugs to test their validity, i.e., medicines that have no nootropic activity must also fail to ameliorate cognitive deficits in a cognition-based task.

We acknowledge the road forward will be long and not without challenges, but the weight of unmet need and a history of rare success signifies it is certainly one worth exploring. Insights from clinical research coupled with a growing toolbox of translational techniques now provide means with which to redefine preclinical development of schizophrenia therapeutics for the twenty-first century.
